# Comparative study on effect of pomegranate peel powder as natural preservative and chemical preservatives on quality and shelf life of muffins

**DOI:** 10.1038/s41598-024-61085-4

**Published:** 2024-05-05

**Authors:** Namrata Ankush Giri, Aditi Bhangale, Nilesh N. Gaikwad, N. Manjunatha, Pinky Raigond, R. A. Marathe

**Affiliations:** ICAR-National Research Centre on Pomegranate, NH-65, Solapur-Pune Highway, Kegaon, Solapur, 413 255 Maharashtra India

**Keywords:** Pomegranate peel, Muffins, Natural preservative, Quality, Microbiology, Antimicrobials, Nutrition

## Abstract

This research aims to investigate the potential of utilizing pomegranate peel powder (PPP) as a natural preservative in muffin preparation. Pomegranate peel is a rich source of bioactive compounds, including phenolics, flavonoids, and tannins, which possess high antioxidant and antimicrobial properties. The In-Vitro antifungal activity of pomegranate peel powder (8% PPP), potassium sorbate (0.1% PS) and calcium propionate (0.5% CP) was assessed against *Penicillium* sp. and *Aspergillus* sp. using poison food technique. The PPP showed the anti-fungal activity by delaying the growth of microorganism on media plate similar to the PS and CP. The effect of utilization of PPP on quality characteristics of muffins were compared with the muffins with chemical preservatives (0.1% PS and 0.5% CP). The viscosity and specific gravity of batter significantly increased from 7.98 to 11.87 Pa s and 1.089–1.398 respectively on addition of 8% PPP. The optical microscopic structure of PPP added batter revealed the decrease in the number of air cells from 24 to 12 with radius range of 6.42–72.72 μm and area range of 511.03–15,383.17 µm^2^. The functional properties of flour with PPP had higher water absorption capacity, foaming stability, emulsification activity and emulsion stability than others. The addition of PPP significantly increase the weight (32.83 g), and decrease the height (31.3 mm), volume (61.43 cm^3^), specific volume (1.67 cm^3^/g) and baking loss (10.19%). The 418.36% increase in fibre content, 14.46% and 18.46% decrease in carbohydrates and energy value was observed in muffin with 8% PPP as compared to control respectively. The total phenols was increased from 0.92 to 12.5 mg GAE/100 g, total tannin from 0.2 to 8.27 mg GAE/100 g, *In-vitro* antioxidant activity by DPPH from 6.97 to 29.34% and In-vitro antioxidant activity by FRAP from 0.497 to 2.934 mg AAE/100 g in muffins added with 8% PPP. The muffin with PPP was softer than control and muffin with 0.1% PS. The addition of PPP resulted to improve in muffin texture but taste slightly bitter. During the storage of muffins at room temperature (27–30 °C), the moisture content of muffin with PPP was reduced from 17.04 to 13.23% which was higher than the rest of the treatments. Similarly, the hardness of sample with PPP was higher than the sample with 0.5% CP, but lowers than control and sample with 0.1% PS throughout the storage period. The results suggest that pomegranate peel powder can be successfully used as a natural preservative in place of chemical preservatives in muffins, to extend the shelf life. This study provides the opportunity to use PPP as functional ingredient and natural preservative in different bakery products.

## Introduction

Muffins are wheat based bakery product, sweet in taste, calorie dense and liked by all age group people and usually consumed throughout the day due to its soft texture and characteristic taste. But, muffins are perishable in nature due to high moisture and having short shelf life^1^. Muffins are dominantly spoiled by molds and chances of contamination takes place in post baking operations as most of the microorganisms killed during baking at high temperature^2^. The major challenges faced in shorter shelf life of muffins are loss in quality due to changes in the moisture content, water activity, storage temperature and microbial growth^3^.

Commercially, the shelf life of muffins is extended using chemical preservatives such as calcium propionate (CP) and potassium sorbate (PS) which inhibits the growth of molds without affecting the product quality. But, their safety and impact on human health is needs to be considered^4^. The consumption of the food products with chemical preservatives for long term may have adverse impact on human health such as toxicity, allergic reaction or the risk of daily over exposure. At present, the food processing sector is looking for the preserving processed products with natural compounds which are safe and satisfy the consumer preference of having healthy and chemical preservative free products in diet. The benefits of using natural ingredients as a natural preservatives are non-toxic and no side health effect. In order to meet the consumer requirement for healthier, safer and functional foods may be satisfied by using the fruit waste/spices essential oils etc. rich in bioactive compounds with anti-microbial properties and anti-oxidant activity may acts as natural preservatives in the processed products^5^. The agro-industrial by-products rich in anti-microbial properties and anti-oxidant activity may be used as safe and non-toxic food additive which acts as natural preservative in muffins. Muffins with label of natural preservatives will be more preferred by the consumers who conscious about the health and well-being^6^.

Pomegranate peel which is 50% part of the fresh fruit weight^7^ is considered as by-product and discarded by pomegranate juice/aril processing industries which cause environmental problems^8^. The antimicrobial activity of pomegranate peel is primarily attributed to its various bioactive compounds, including polyphenols, tannins, flavonoids, and alkaloids^9^. The high level of ellagitannin is responsible for the antimicrobial properties of the pomegranate peel^10^. These compounds possess antimicrobial properties that can inhibit the growth and proliferation of various microorganisms, including bacteria, fungi, and viruses^11^.

Pomegranate peel powder (PPP) has been explored as a natural preservative in various food products due to its antimicrobial properties^12^. The natural preservative action of pomegranate peel was studied for the improvement in the shelf life of the food products due to the presence of bioactive compounds^13,14^. It can help inhibit the growth of spoilage-causing microorganisms, extend shelf life, and maintain the quality and safety of the food. It is not only rich in bioactive compounds but also rich in fibre content. The dietary fiber content of 12 different cultivars of pomegranate peel was reported to be ranging between 33.10 and 62.09% by Hasnaoui et al.^15^. The utilization of PPP in food products may not only enhance the shelf life but will also improve the nutritional value in terms of the fibre content and having high antioxidant activity. Many researchers have also used PPP as a source of fibre for the fortification of cookies^16,17^, bread^18,19^, and cake^20,21^. The increase in oxidative stability of food products due to the addition of PPP or extract was also reported by Abozed et al.^22^.

The PPP can be used as a functional ingredient not only to enhance the nutritional profile but also to improve the shelf stability of muffins. Hence, in the present investigation, a study was carried out on the effect of PPP as a natural preservative on quality attributes and shelf stability of the muffins in comparison with chemical preservatives such as PS and CP.

## Materials and methods

### Materials

The fully matured pomegranate fruits (Bhagawa Cv.) were harvested from the experimental block of Indian Council of Agricultural Research (ICAR)-National Research Centre on Pomegranate (NRCP), Solapur and harvested pomegranate fruits were transferred to the Post-Harvest Technology laboratory of NRCP. Raw materials such as refined wheat flour, refined sugar, refined oil, milk, egg, sodium bicarbonate, and vanilla essence were procured from the local market of Solapur City. Calcium propionate and potassium sorbate (HiMedia Laboratories Pvt. Ltd., Mumbai) were also procured. The chemicals used were analytical and food grade.

### Methods

#### Pomegranate peel powder preparation

The disease-free, good-quality pomegranate fruits were separated and washed with sodium hypochlorite (80 ppm, pH 7) followed by normal water wash for surface disinfection. Fruits were cut and arils separated manually and peels of fruits were cut into pieces. These were dried at 50 °C in a cabinet tray drier (MAC-MSW 216, Macro Scientific Works) till the moisture content of the peel reaches approx. to 5%. The pomegranate dried peels were pulverized into powder using a hammer mill to pass through 60 mesh sieve of BSS standard to obtain a particle size of 250 µm and stored at a low temperature (5 °C) until further use^23^.

#### Assay of in-vitro antifungal activity

The antifungal activity of chemical preservatives (CP and PS) and PPP against *Aspergillus* sp. (Accession No. OP01089) and *Penicillium* sp. (Accession No.OP01090) which are responsible for the spoilage of muffins was determined using poison food technique^24^. Briefly, the PPP (8%), PS (0.1%), and CP (0.5%) were incorporated into potato dextrose agar (PDA) before the inoculation of target fungi. The plates without the addition of PPP and chemical preservatives served as a control to compare the growth inhibition. The PDA plates with preservatives were inoculated with target fungi [*Aspegillus* sp. (Accession No. OP01089) and *Penicillium* sp*.* (Accession No.OP01090)] by spreading 0.1 ml of spore suspension. Later, the plates were incubated at 25 °C. The inhibitory action against the spoilage fungi was recorded based on the total number of colonies that appeared at different time intervals. The level of 0.5% CP and 0.1% PS was considered as per the maximum permissible limit by FSSAI as preservatives in bakery products. Moreover, the level of 8% PPP was selected based on a preliminary experiment conducted on optimization of the maximum level of addition of PPP in muffins without altering the sensory characteristics.

#### Muffin formulation

The muffins were prepared as per the formulations given in Table 1. The four treatments of muffins were prepared viz. control (without preservative), chemical preservative (muffins with 0.5% CP and 0.1% PS) and natural preservative (muffin with 8% PPP; already standardized). The method was followed as described by Goswami et al.^25^ for muffin preparation. Among all the listed ingredients (Table 1), fresh egg white and yolk were used in the proportion of 2.7:1 in the formulation of the muffins. The ingredients mentioned in Table 1 were mixed properly in a spiral mixer and the batter was poured into a muffin tray and baked at 200 °C for 20 min. in the preheated baking oven. The muffins were cooled at room temperature (27–30 °C) and packed in high-density polyethylene bags (HDPE) until further analysis. Muffins with 8% PPP required to add extra 8% liquid phase in the form of milk due to fibrous nature of PPP which tends to maximum water absorption and high viscous batter preparation^23^. The preliminary experiment was carried out to optimize the level of PPP (0–10%) in muffins. Muffins with 8% PPP were selected based on the textural properties, shelf stability with respect to oxidative and microbial stability. Moreover, muffins with 10% PPP was not sensorial acceptable with lower score for texture and taste as compared to muffins with 8% PPP^23^.

**Table 1:** The formulation of muffins with pomegranate peel powder and chemical preservatives.

Ingredients	Control	Chemical preservative		Natural preservative
		0.5% CP	0.1% PS	8% PPP
Refined wheat flour (%)	26	25.5	25.9	18
Pomegranate peel powder (PPP) (%)	0	0	0	8
Calcium propionate (CP) (%)	0	0.5	0	0
Potassium sorbate (PS) (%)	0	0	0.1	0
Edible oil (%)	12	12	12	12
Refined sugar (%)	26	26	26	26
Milk (%)	13	13	13	21 (8% extra liquid phase)
Egg (%)	22 (Egg white: Yolk, 2.7:1)	22 (Egg white: Yolk, 2.7:1)	22 (Egg white: Yolk, 2.7:1)	22 (Egg white: Yolk, 2.7:1)
Sodium bicarbonate (%)	1	1	1	1
Edible salt (%)	0.1	0.1	0.1	0.1
Vanilla Essence (ml)	0.5	0.5	0.5	0.5

#### Physical properties of muffin batter

The viscosity and specific gravity of the muffin batter were evaluated according to Soumya et al.^1^. The viscosities of all samples were measured using spindle no.7, at 20 rpm for 5 s of Anton Paar (Model ViscoQC 100R) at room temperature. Moreover, the specific gravity of the batter was determined as the ratio of weight of a known volume of the batter to the weight of an equal volume of water. The colour value of muffin batter in terms of L*, a*, and b* values was measured using Colour Difference Meter (Lab Scan XE, Hunter Color Lab).

#### Optical microscopy of muffin batter

The effect of the use of PPP, CP and PS on the air bubble expansion in muffin batter was investigated by using a Nikon (Eclipse 90 I, Kawasaki, Japan) light microscope. One drop of freshly prepared muffin batter was placed on a microscopic slide and covered with a covering slip. The samples were observed under a microscope in clear field mode with a 10× magnification. Microscopic images of batter were captured with a digital camera mounted on a microscope^26^.

#### Functional properties of the muffin premix

The functional properties of muffin premix includes control (refined wheat flour), 0.5% CP (refined wheat flour + calcium propionate), 0.1% PS (refined wheat flour + potassium sorbate) and 8% PPP (refined wheat flour + pomegranate peel powder) were evaluated according to the methods described by Giri et al.^27^. The functional properties includes water absorption capacity (%), oil absorption capacity (%), bulk density (g/ml), tapped density (g/ml), dispersibility (ml), foaming capacity (%), foaming stability (%), emulsification activity (%), emulsification stability (%) and swelling capacity (ml).

##### Water absorption capacity (WAC)

One gram of the sample was mixed with 10 ml of water for 1 min. and it was allowed to stand for 30 min. and centrifuged at 1200 × *g* for 30 min. (Sigma laboratory centrifuge 3 K 18, Germany). The volume of free water was recorded directly from the centrifuge tube.

$${\text{WAC }}\left( \% \right) \, = \frac{{\left( {{\text{Amount of water added }} - {\text{ Free water}}} \right) \, \times {\text{ Density of water }} \times { 1}00}}{{{\text{Weight of sample }}\left( {\text{g}} \right)}}$$

##### Oil absorption capacity (OAC)

Similar to WAC, 1 g of the sample was mixed with 10 ml refined corn oil in a centrifuge tube and allowed to stand for 1 h then centrifuged at 1600 × *g* for 20 min (Sigma laboratory centrifuge 3 K 18, Germany). The volume of free oil was recorded and decanted. Oil absorption capacity was expressed as millilitre of oil bound by 100 g dried sample.

##### Bulk density

Ten gram of the sample was weighed into 50 ml graduated measuring cylinder and was gently tapped until there was no further change in the sample level after filling to the mark on the cylinder. The volume of the sample was recorded.

$${\text{Bulk density }}\left( {{\text{g}}/{\text{ml}}} \right) \, = \frac{{{\text{Weight of sample }}\left( {\text{g}} \right)}}{{\text{Volume of sample after tapping}}}$$

##### Tapped density

After obtaining the volume for bulk density, the cylinder was tapped 50 times and the corresponding volume was noted. The tapped density was expressed as the weight of sample per volume of sample.

##### Dispersibility

Ten grams of sample was suspended in 200 ml measuring cylinder and distilled water was added to reach the 100 ml mark. The set-up was stirred vigorously and allowed to settle for 3 h. The volume of settled particles was recorded and dispersibility of sample was determined.

$${\text{Dispersibility }} = { 1}00 \, - \, \left( {\text{Volume of settled particle}} \right)$$

**Foaming capacity and stability:** It was determined by mixing 2 g of sample with 50 ml water in 100 ml measuring cylinder followed by shaking the suspension to foam, and total volume of foam after 30 s was noted and foaming capacity was calculated as percent increase in volume after 30 s. However, foam stability was recorded as volume of foam after 1 h of whipping the suspension.

##### Emulsification activity

A known quantity (7 g) of sample preparation was suspended in 100 ml distilled water and added 100 ml of oil followed by emulsification of mixture in a homogenizer at 10,000 rpm for 1 min. The emulsion obtained was centrifuged at 1300 rpm for 5 min.

$${\text{Emulsification activity }}\left( \% \right) \, = \frac{{{\text{Height of emulsion layer}} \times {1}00}}{{\text{Total emulsion layer}}}$$

##### Emulsification stability

A known quantity (7 g) of sample preparation was suspended in 100 ml distilled water and added 100 ml of oil followed by emulsification of mixture in a homogenizer at 10,000 rpm for 1 min. Heat emulsion at 80 °C for 30 min and centrifuge at 1300 rpm for 5 min.

$${\text{Emulsification stability }}\left( \% \right) \, = \frac{{{\text{Height of remaining emulsion}} \times {1}00}}{{\text{Total height of emulsion}}}$$

#### Physical properties of muffins

Height, weight, volume, specific volume and baking loss of muffins were measured^28^. The colour value of muffins in terms of L*, a*, and b* value was measured using Colour Difference Meter (Lab Scan XE, Hunter Color Lab). The total colour difference (ΔE) between control and muffins with chemical and natural preservative were also calculated^29^ as follows:

$$\Delta {\text{E }} = \left[ {\left( {\Delta {\text{L}}*} \right)^{{2}} + \, \left( {\Delta {\text{a}}*} \right)^{{2}} + \, \left( {\Delta {\text{b}}*} \right)^{{2}} } \right]^{{{1}/{2}}}$$

Browning index (BI) was calculated to know the purity of brown colour of muffin crust. The brown colour formation is due to non-enzymatic browning of muffin crust^30^. The BI for muffin crust was calculated using formula^31^.

$${\text{BI}} = \frac{{\left[ {{1}00\left( {{\text{x}} - 0.{31}} \right)} \right]}}{{0.{172}}}$$

where,

$${\text{x }} = \frac{{\left( {{\text{a }} + { 1}.{\text{75 L}}*} \right)}}{{\left( {{5}.{\text{645L }} + {\text{ a}}_{0} {-}{ 3}.0{\text{12 b}}} \right)}}$$

where, a_0_ = ‘a’ value of muffin batter.

The texture profile analysis (TPA) of the muffins samples were measured using Texture analyzer (TA XT Plus, Stable Microsystem UK). A sample size of 2 × 2 × 2 (cm^3^) from crumb of each muffins was taken for the measurement of texture profile analysis in terms of hardness (g), adhesiveness (g s), springiness (mm), cohesiveness, gumminess (g), chewiness (mJ) and resilence. A cylindrical probe (P/45) having 45 mm diameter with 50% strain at a speed of 1 mm/s was used and a double compression test was performed as per the procedure explained by Martínez-Cervera et al.^32^.

#### Proximate composition of muffins

The proximate composition includes moisture (%), protein (%), fat (%), fiber (%), and ash (%) of muffin samples were determined by AOAC methods^33^. Ash was performed at 550 °C for 2 h (g ash/100 g sample). Protein (g protein/100 g sample) was analyzed according to the Kjeldahl method. Fat (g fat/100 g sample) was calculated by weight loss after a 6-cycle extraction with petroleum ether in a Soxhlet apparatus. Carbohydrate content (%) was evaluated by difference method and energy value (kcal/100 g) measured as:

$${\text{Energy value }}\left( {{\text{kcal}}/{1}00{\text{g}}} \right) \, = \, (\% {\text{ carbohydrates}} \times {4}) \, + \, (\% {\text{ protein}} \times {4}) \, + \, (\% {\text{ fat}} \times {9})$$

#### Bioactive compounds and in-vitro antioxidant activity of muffins

A methanolic extract of samples were prepared by taking 1 g sample in 10 ml of methanol and kept for overnight, followed by orbital shaking for 3 h at 300 rpm, centrifugation (10,000 rpm for 10–15 min) and the resultant supernatant was further used for the estimation of bioactive components. The bioactive compounds such as total phenols was evaluated using Folin–Ciocalteu (FC) reagent method explained by Abirami et al.^34^, total tannin content were also determined by the same method used for total phenols with slight modifications^35^. The In-vitro antioxidant activity was assessed by FRAP method as explained by Benzie and Strain^36^ and DPPH method^37^.

#### Scanning images of horizontal section of muffin

The total number of pores or bubbles of muffins were measured according to Martínez-Cervera et al.^32^ with slight modifications. The horizontal cut of the muffin from the base were taken and images of the freshly cut surface of crumb were captured using a flatbed scanner (hp Scanjet 4850). Image processing was performed using Adobe Photoshop CS. Ink software. The images were converted to an 8-bit grey scale for the measurement of number of bubbles within an area of 0.01 m^2^.

#### Sensory characteristics of muffins

The sensory evaluation of muffins prepared with PPP, CP, PS and control sample were evaluated by a panel of 50 semi-trained members with the help of a sensory score card. Each formulation was assigned a random 3 digit number. Sensory evaluation was performed using a Nine point Hedonic scale. The Hedonic scale was in the following sequence (like extremely—9, like very much—8, like moderately—7, like slightly—6, neither like nor dislike—5, dislike slightly—4, dislike moderately—3, dislike very much—2, dislike extremely—1). The panel was provided with two pieces of muffins for every experimental sample and asked to score them for different sensory attributes. Water and unflavoured rice puffs were also provided to the panellist for cleansing their palate in between the evaluation of the different samples^38^.

### Storage studies

Muffins prepared with PPP, chemical preservative (CP and PS) and control was prepared in bulk for the shelf life evaluation. These samples were packed in high density polyethylene bags and stored at room temperature (27–30 °C) for the period of 14 days.

The muffins with and without preservatives were evaluated for moisture content,and texture profile analysis during the storage period^39^.

### Statistical analysis

The results obtained are expressed as standard deviation of analysis performed in triplicate for each parameters except sensory characteristics (n = 50). A one-way analysis of variance and Duncan’s test were used to establish the significance of differences among the mean values at the 0.05 significance level. The statistical analyses were performed using SPSS 16.0 software^40^.

## Results and discussions

### In-vitro antifungal activity

**Figure 1:**
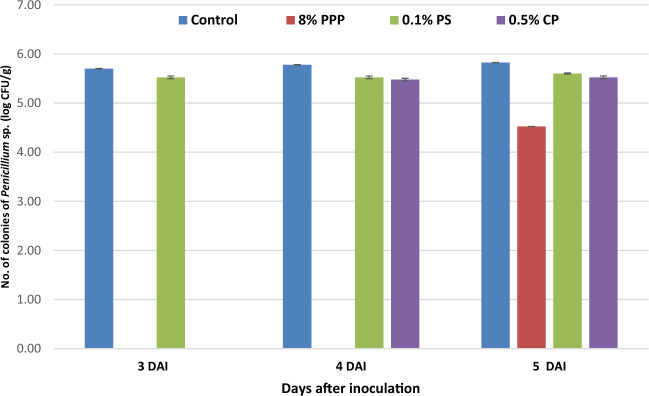
Antifungal activity of control, pomegranate peel powder (PPP), potassium sorbate (PS) and calcium propionate (CP) against *Penicillium sp*.

The In-vitro antifungal activity was performed for PPP and compared with two chemical preservatives widely used in the food industry, CP and PS against *Penicillium* sp. and *Aspergillus* sp. using poison food techniques. The results demonstrated that PPP showed significantly strong inhibition against *Penicillium* sp*.* as compared to PS and CP from 0 to 5 days after inoculation (Fig. 1). The total number of *Penicillium* sp. colonies appeared on 3rd day after inoculation in control as 5.70 log CFU/g and 0.1% PS as 5.52 log CFU/g. Moreover, the colonies observed on the 4th day after inoculation in the case of 0.5% CP as 5.47 log CFU/g and 5th day after inoculation in the case of PPP as 4.51 log CFU/g. It was investigated that, the PPP had the ability to delay the growth of *Penicillium* sp*.* followed by CP and PS. The growth of *Penicillium* sp*.* can be suppressed in food products containing PPP for a longer duration as compared to chemical preservatives. The ethanol extract of pomegranate peel showed effective against controlling the growth of *A. niger* and *Penicillium* sp. as compared to aqueous extract^41^.

**Figure 2:**
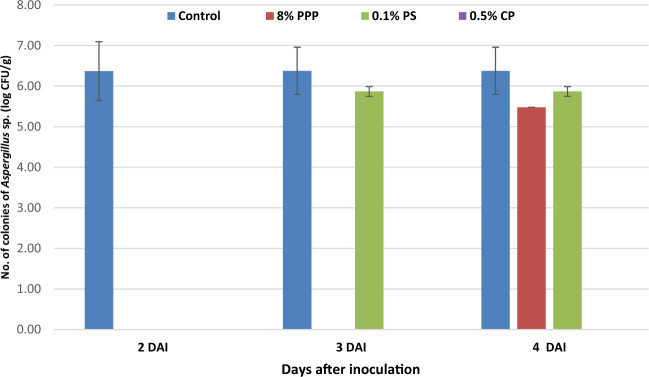
Antifungal activity of control, pomegranate peel powder (PPP), potassium sorbate (PS) and calcium propionate (CP) against *Aspergillus* sp.

The In-Vitro antifungal activity of PPP and chemical preservatives against *Aspergillus* sp. was also studied (Fig. 2) and PPP was found effective to prevent the growth of *Aspergillus* sp. as equal to the PS. However, CP had complete inhibitory action against *Aspergillus* sp. The total numbers of colonies were reported on 3rd days after inoculation as 5.47 and 5.86 log CFU/g for media plates with PPP and PS respectively. Whereas, the control plate had count of 6.36 log CFU/g on 2nd days after inoculation. Naseem et al.^42^ reported the highest sensitivity of pomegranate peel against *Aspergillus parasiticus* among all the fungi.

The In-vitro antifungal activity of PPP and chemical preservatives showed that, PPP had the ability to inhibit the growth of *Penicillium* sp. and *Aspergillus* sp. which are responsible for the spoilage of muffins for the defined period. PPP may be used as natural preservative to extent the shelf life of muffins due to its ability to supress the growth of muffin spoilage microorganisms. The presence of polyphenols and punicalagins are responsible for the pomegranate antifungal activities were reported by Mertens-Talcott et al.^43^ and Seeram et al.^44^. The antifungal activity of pomegranate peel is responsible for cell death due to formation of pore-like structure by punicalagin. The glucoside formed the inner wall, and ellagic acid formed the outer wall of microbial cell, and all these combine change the physiological transmembrane gradients of microbial cell leads to cell death^45^. Higher the level of total phenol content, high value for antifungal activity in 21 different pomegranate genotype was reported by Rongai et al.^46^.

### Physical properties of muffin batter

The effect of addition of 8% PPP and chemical preservatives (0.1% PS and 0.5% CP) on batter characteristics is given in Table 2. Addition of PPP showed a significant increase in batter viscosity and specific gravity as compared to batter with chemical preservatives and control sample. There is no significant difference between control and batter with chemical preservatives with respect to viscosity and specific gravity.

**Table 2:** Physical properties of muffin batter with pomegranate peel powder and chemical preservatives.

Treatments	Viscosity (Pa s)	Specific gravity	L*	a*	b*	ΔE
Control	7.98 ± 0.01^b^	1.089 ± 0.02^b^	83.35 ± 0.05^b^	3.31 ± 0.03^b^	32.81 ± 0.01^b^	0 ± 0
8% PPP	11.87 ± 0.03^a^	1.398 ± 0.02^a^	41.20 ± 0.05^c^	11.41 ± 0.03^a^	41.06 ± 0.01^a^	43.70 ± 0.03^a^
0.1% PS	7.87 ± 0.05^b^	1.045 ± 0.01^b^	83.43 ± 0.05^b^	3.27 ± 0.03^b^	32.67 ± 0.03^b^	0.16 ± 0.02^c^
0.5% CP	7.96 ± 0.01^b^	1.064 ± 0.02^b^	84.34 ± 0.05^a^	2.9 ± 0.02^c^	30.4 ± 0.03^c^	2.63 ± 0.02^b^

Values are expressed as mean ± SD of three independent determinations. Values in columns followed by the same letter are not significantly different at p ≤ 0.05 as measured by Duncan’s test.

PPP, pomegranate peel powder; PS, potassium sorbate; CP, calcium propionate.

The muffin batter with PPP had higher viscosity due to the high concentration of dietary fibre such as pectin, lignin, and cellulose in pomegranate peel leading to higher absorption of water which resulted into increase in the batter viscosity. The similar observations were reported by Topkaya and Isik^21^ and Oliveira et al.^47^. The specific gravity of PPP is 0.68 which is quite higher than refined wheat flour (0.66) (data not shown) and thus addition of PPP resulted in low air incorporation and less air bubble formation and lower the specific gravity of batter^23^. Goswami et al.^25^ reported a significant increase in the specific gravity of batter when there is an increase in the level of barnyard millet flour which is rich in fiber included the formulation.

The colour value of muffin batter samples in terms of ‘L*’, ‘a*’ and ‘b*’ value were measured (Table 2) and muffin batter with 8% PPP had significantly highest ‘a*’ (redness) and ‘b*’ (yellowness) values as compared to rest of the treatments. The ‘L*’ (lightness) value was significantly lower (41.20) for sample with 8% PPP. The colour value of refined wheat flour (L* = 93.46, a* = 0.37, b* = 9.45) and PPP (L* = 61.76, a* = 13.20, b* = 36.88) showed a higher lightness value for refined wheat flour, whereas redness and yellowness value for PPP. Therefore, the muffin batter with PPP showed high value for ‘a*’ and ‘b*’. The total colour change of batter with respect to control sample is presented by ‘ΔE’. Negligible colour changes were observed in muffin batters with chemical preservatives (0.16–2.63) as compared to batter with PPP (43.70) in comparison with control sample. The change in muffin batter colour on addition of lemon grass, clove and cinnamon powder was reported by Soumya et al.^1^.

### Optical microscopy of muffin batter

The number of air cells and its size in batter is indirectly related to the porosity and sponginess of the baked muffins. The entrapment of the air in the batter is the result of the number of bubbles/air cells formed. The muffin batter added with 8% PPP showed least number of air cells (12) as compared to control (24) and batter with chemical preservatives (21–22). It may be due to the fibrous nature of PPP and replacement of refined wheat flour which leads to the gluten dilution. The viscoelastic nature of gluten protein has the ability to entrap the air bubbles. The maximum number of air bubbles, caused maximum porosity of the muffins^48^. There is no significant difference between the number of air cells, its radius and area range for the batter of control sample and sample with chemical preservatives. The air cell radius range for batter of different treatments as control (17.7–52.52 μm), batter with 0.1% PS (18.8–49.62 μm) and 0.5% CP (15.72–73.48 μm). But, the incorporation of PPP resulted to the decrease in number of air cells, 6.42–72.72 μm as compared to control and batter with chemical preservatives. The area range of batter air cell with PPP was in the range of 511.03–15,383.17 µm^2^ which was lower than the control sample (1510.87–8575.1 µm^2^), batter with 0.1% PS (1555.31–8260.12 µm^2^) and 0.5% CP (691.39–16,245.77 µm^2^). Although the number of air cells were less in batter with PPP but the air cell radius range and its area range were found greater than for the batter sample of control and batter with 0.1% PS.

**Figure 3:**
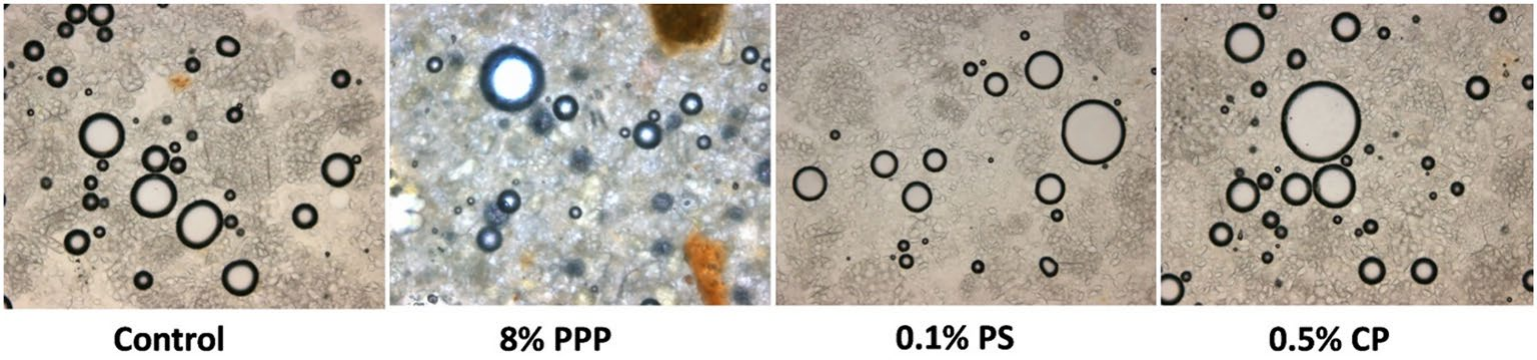
Optical microscopy images for batter samples of control (without preservatives), batter with 8% PPP, 0.1% PS and 0.5% CP.

The optical microscopic images of muffin batter showed that, the appearance of air cell were smooth round, distinctive and globular in batter of control sample, batter with 0.1% PS and 0.5% CP (Fig. 3). However, the air cell of rough surface, dense and compact were observed in batter with PPP. This could be due to the high fiber content of PPP which disrupt the bubbles formation in the batter. The significant changes in the muffin batter attributes due to the fortification of fiber rich red capsicum pomace powder is reported by Nath et al.^49^.

### Functional properties of the muffin premix

The effect of addition of PPP and chemical preservatives on functional properties of muffin premix (blend of refined wheat flour + 8% PPP/0.5% CP/0.1% PS) were evaluated and compared with control sample (refined wheat flour) (Table 3). Muffin premix containing PPP had significantly higher water absorption capacity (91.23%), foaming stability (2.68%), emulsification activity (52.12%) and emulsion stability (48.28%) as compared to control and premix with chemical preservatives. Whereas, no significant difference were observed for functional properties of muffin premix of control and premix with chemical preservatives. Its mean that, the addition of 0.5% CP and 0.1% PS in refined wheat flour for preparation of muffins doesn’t had any significant effect on functional properties, but the addition of 8% PPP affect the functional properties. The high fiber content of pomegranate peel powder is responsible for the higher water absorption capacity. The similar results are also quoted by Topkaya and Isik^21^ and Giri et al.^23^. The high water holding capacity of carrot fiber added muffin flour is reported by Psimouli and Oreopoulou^48^.

**Table 3:** Functional properties of the muffin premix with PPP, chemical preservatives and control.

Treatments	Water absorption capacity (%)	Oil absorption capacity (%)	Bulk density (g/ml)	Tapped density (g/ml)	Dispersibility (ml)	Foaming capacity (%)	Foaming stability (%)	Emulsification activity (%)	Emulsion stability (%)	Swelling capacity (ml)
Control	89.02 ± 0.02^b^	78.34 ± 0.01^a^	0.53 ± 0.03^a^	0.70 ± 0.03^a^	71.00 ± 0.05^a^	12.05 ± 0.05^a^	1.83 ± 0.05^b^	49.57 ± 0.03^b^	46.27 ± 0.03^b^	17.00 ± 0.05^a^
8% PPP	91.23 ± 0.03^a^	76.26 ± 0.03^b^	0.47 ± 0.02^b^	0.60 ± 0.03^b^	66.00 ± 0.05^b^	10.64 ± 0.03^b^	2.68 ± 0.03^a^	52.12 ± 0.02^a^	48.28 ± 0.05^a^	9.00 ± 0.03^b^
0.1% PS	89.14 ± 0.02^b^	78.87 ± 0.02^a^	0.53 ± 0.03^a^	0.69 ± 0.02^a^	70.00 ± 0.03^a^	12.89 ± 0.05^a^	1.94 ± 0.03^b^	49.87 ± 0.03^b^	46.23 ± 0.05^b^	17.00 ± 0.05^a^
0.5% CP	89.32 ± 0.05^b^	78.23 ± 0.05^a^	0.52 ± 0.05^a^	0.68 ± 0.03^a^	71.00 ± 0.02^a^	12.27 ± 0.03^a^	1.86 ± 0.05^b^	49.88 ± 0.05^b^	46.36 ± 0.05^b^	17.00 ± 0.05^a^

Values are expressed as mean ± SD of three independent determinations. Values in columns followed by the same letter are not significantly different at p ≤ 0.05 as measured by Duncan’s test.

PPP, pomegranate peel powder; PS, potassium sorbate; CP, calcium propionate.

The functional properties gives an idea about the amount of oil and water phase to be used based on the fortification of new ingredients in the formulation. Also, the behaviour of the materials while processing into products^45^.

### Physical properties of muffins

From the data mentioned in Table 4, it was observed that, the significant difference in the physical properties of muffins was taken place due to addition of PPP whereas, muffins with chemical preservatives showed non-significant difference as compared to control. The weight of muffins containing PPP was significantly higher (32.83 g) than muffins with chemical preservatives and control. The addition of PPP also leads to decrease in the height (31.3 mm), volume (61.43 cm^3^), specific volume (1.67 cm^3^/g) and baking loss (10.19%) of muffins when compared to control and muffins with chemical preservatives. The per cent baking loss was 10.19% in muffins with PPP which was significantly lower than muffins with chemical preservatives and control. It could be due to high moisture holding capacity of fiber present in PPP which prevent the loss of moisture during baking and resulted to decrease in baking loss and increase in weight^23^. The presences of fiber in PPP also resulted to the gluten dilution in the muffins and interfere in air entrapment in the batter which is desirable for the increase in the height, volume and specific volume of the muffins. The similar findings are reported by Psimouli and Oreopoulou^48^ in carrot fiber enriched cake; Topkaya and Isik^21^ in pomegranate peel supplemented muffin cake; Gadallah et al.^50^ in cupcake with pomegranate peel powder and Giri et al.^23^ in muffins fortified with pomegranate peel powder.

**Table 4:** Physical properties of muffins with pomegranate peel powder and chemical preservatives.

Treatments	Weight (g)	Height of muffins (mm)	Volume (cm^3^)	Specific Volume (cm^3^/g)	Baking loss (%)
Control	31.46 ± 0.05^b^	33.9 ± 0.03^a^	62.84 ± 0.05^a^	1.99 ± 0.02^a^	11.23 ± 0.02^a^
8% PPP	32.83 ± 0.05^a^	31.3 ± 0.05^b^	61.43 ± 0.01^b^	1.67 ± 0.05^b^	10.19 ± 0.05^b^
0.1%PS	31.49 ± 0.01^b^	33.5 ± 0.02^a^	62.43 ± 0.01^a^	1.98 ± 0.01^a^	11.45 ± 0.05^a^
0.5% CP	31.27 ± 0.05^b^	33.8 ± 0.01^a^	62.65 ± 0.02^a^	1.98 ± 0.03^a^	11.63 ± 0.05^a^

Values are expressed as mean ± SD of three independent determinations. Values in columns followed by the same letter are not significantly different at p ≤ 0.05 as measured by Duncan’s test.

PPP, pomegranate peel powder; PS, potassium sorbate; CP, calcium propionate.

The colour values of the muffins were expressed in terms of ‘L*’, ‘a*’, ‘b*’ and ‘ΔE’. From the Table 5 it was clear that, the addition of PPP in muffin significantly affected on the colour value of crust and crumb of muffins when compared with colour value of control sample. Whereas, the colour value of muffins added with chemical preservatives had almost similar colour value with control sample.

**Table 5:** Colour value of muffins with pomegranate peel powder and chemical preservatives.

Treatments	L*	a*	b*	ΔE	BI
Muffin crust					
Control	61.8 ± 0.03^a^	16.73 ± 0.02^a^	45.27 ± 0.03^a^	0 ± 0	215.81 ± 0.05^a^
8% PPP	27.03 ± 0.03^b^	4.52 ± 0.02^b^	3.75 ± 0.05^b^	55.51 ± 0.01^a^	152.64 ± 0.03^b^
0.1% PS	61.44 ± 0.05^a^	15.53 ± 0.03^a^	44.24 ± 0.05^a^	1.62 ± 0.05^b^	216.84 ± 0.05^a^
0.5% CP	58.89 ± 0.02^a^	17.29 ± 0.05^a^	43.49 ± 0.01^a^	3.45 ± 0.03^b^	204.34 ± 0.05^a^

	L*	a*	b*	ΔE	
Muffin crumb					
Control	72.8 ± 0.03^a^	6.11 ± 0.02^c^	36.69 ± 0.05^a^	0 ± 0	
8% PPP	21.34 ± 0.03^b^	9.33 ± 0.05^a^	14.68 ± 0.05^b^	56.06 ± 0.03^a^	
0.1% PS	72.72 ± 0.05^a^	8 ± 0.03^b^	40.93 ± 0.05^a^	4.64 ± 0.03^b^	
0.5% CP	75.24 ± 0.05^a^	4.94 ± 0.05^d^	35.86 ± 0.03^a^	2.83 ± 0.05^b^	

Values are expressed as mean ± SD of three independent determinations. Values in columns followed by the same letter are not significantly different at p ≤ 0.05 as measured by Duncan’s test.

PPP, pomegranate peel powder; PS, potassium sorbate; CP, calcium propionate; BI, Browning index.

**Figure 4:**
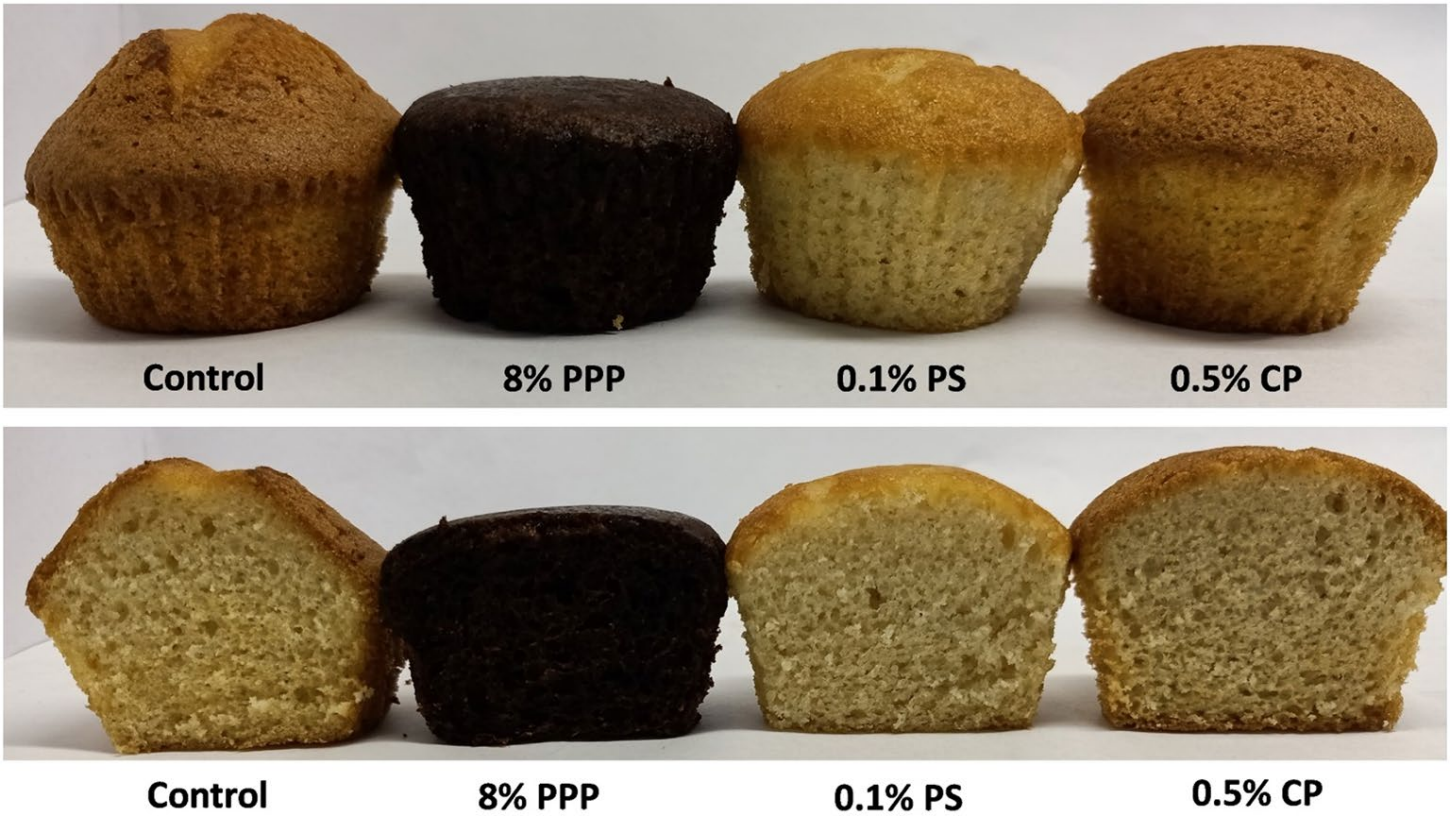
Muffins with pomegranate peel powder and chemical preservatives.

The L* (27.03), a* (4.52), and b* (3.75) value for muffin crust with PPP was lower than the colour values for crust of the rest of the samples. The total colour change (ΔE) value was very high for the muffin crust with PPP (55.51), and low for muffin with 0.5% CP (3.45) and 0.1% PS (1.62) which represents the drastic difference in the colour of the muffin crust of PPP added muffin in comparison with control (Fig. 4). The browning index (BI) of muffin crust was calculated which represents the intensity of brown colour formation on crust of muffins due to non-enzymatic reaction specially Maillard reaction and caramelization of sugars during baking^51^. The BI of muffin with PPP was 152.64 which were significantly lower than BI of control (215.81). The muffin with chemical preservatives showed higher BI as compared to muffin with PPP. It may be due to the high protein and sugar content of refined wheat flour as compared to PPP, which are responsible for the brown colour formation during baking.

The addition of PPP had significant effect on the colour value of muffin crumb when compared with crumb colour value of muffin with chemical preservatives and control. The significant increase in ‘a*’ value (9.33), and decrease in ‘L*’ (21.34) and ‘b*’ (14.68) value was observed due to the addition of PPP in muffins. It is evident from the result that, the total colour change (ΔE) in muffin crumb was highest (56.06) recorded in sample with PPP. The addition of PPP in muffin resulted to development of dark chocolate colour of the crust as well as crumb (Fig. 4) which leads to major total colour change with respect to control. The presence of granatonine pigment^52^ in pomegranate peel is responsible for the darker colour of muffins fortified with 8% PPP. The significant decrease in the L* and b* value of both crust and crumb of muffins on increase in the level of addition of PPP was reported by Topkaya and Isik^21^. The appearance of darker colour in muffins crust and crumb fortified with red capsicum pomace powder is reported by Nath et al.^49^.

### Texture profile analysis of muffins

The texture profile analysis (TPA) of fresh muffin samples with PPP, chemical preservatives and control were evaluated and shown in Table 6. From the textural data, most important parameter for muffin is hardness. The hardness of muffin with 8% PPP (382.12 g) significantly similar as that of sample with 0.5% CP (313.86 g) (Table 6). The addition of extra liquid phase in the formulation of muffins with 8% PPP makes it softer than the rest of the treatments (Table 1). In present study, the desirable characteristic of muffin with PPP is softness as compared to control and sample with chemical preservatives, due to use of extra liquid phase in the formulation. Otherwise, the fruit by-products rich in dietary fiber is responsible for high water and oil binding capacity and increased in hardness of the product. Our findings are in concur with statement made by Elleuch et al.^53^.

**Table 6:** Texture profile analysis of fresh muffins with pomegranate peel powder and chemical preservatives.

Treatments	Hardness (g)	Adhesiveness (g s)	Springiness (mm)	Cohesiveness	Gumminess (g)	Chewiness (mJ)	Resilence
Control	685.3 ± 0.01^a^	− 0.991 ± 0.02^c^	0.874 ± 0.01^b^	0.637 ± 0.01^b^	436.8 ± 0.03^a^	381.95 ± 0.01^a^	0.266 ± 0.03^b^
8% PPP	382.12 ± 0.01^c^	− 0.717 ± 0.03^b^	0.908 ± 0.01^a^	0.557 ± 0.03^c^	212.9 ± 0.03^c^	193.32 ± 0.05^c^	0.261 ± 0.03^b^
0.1% PS	517.53 ± 0.05^b^	− 1.58 ± 0.05^d^	0.883 ± 0.05^b^	0.679 ± 0.05^a^	351.4 ± 0.05^b^	310.26 ± 0.05^b^	0.283 ± 0.02^a^
0.5% CP	313.96 ± 0.05^c^	− 0.503 ± 0.05^a^	0.882 ± 0.05^b^	0.641 ± 0.05^b^	201.16 ± 0.03^d^	177.47 ± 0.02^d^	0.27 ± 0.01^a^

Values are expressed as mean ± SD of three independent determinations. Values in columns followed by the same letter are not significantly different at p ≤ 0.05 as measured by Duncan’s test.

PPP, pomegranate peel powder; PS, potassium sorbate; CP, calcium propionate.

The addition of PPP also reduced the cohesiveness, gumminess and chewiness of muffins as compared to control and muffins with chemical preservatives. But, the springiness value was higher for the muffin with PPP. Resilience is textural properties which measure the time require to regain the original shape of the product after compression. Lower the value, higher the time required. Although, there is a statistical difference for the resilience value for muffins with PPP, chemical preservatives and control, but numerically the values are in the range of 0.261–0.283^23^.

### Proximate composition of muffins

The proximate compositions of the muffins are depicted in Table 7. The addition of PPP resulted to increase in crude fibre (2.45–12.7%), ash (1.70–3.32%) and moisture (15.32–17.04%) content as compared to control sample. In context to the fiber content, the incorporation of 8% PPP in muffins increased the fiber content 6 times as compared to control and sample with CP and PS. Overall, 14.46% and 18.46% decrease in carbohydrates and energy value was observed in muffin with 8% PPP as compared to control respectively. Whereas, the addition of chemical preservatives in muffins doesn’t show major changes in the proximate composition as compared to the control sample. Because, CP and PS does not contribute to the nutritional value as these are the preservatives.

**Table 7:** Proximate composition of muffins with PPP and chemical preservatives.

Treatment	Moisture (%)	Protein (%)	Fats (%)	Crude Fibre (%)	Ash (%)	Carbohydrates (%)	Energy value (kcal/100 g)
Control	15.32 ± 0.03^b^	7.37 ± 0.03^a^	11.89 ± 0.02^a^	2.45 ± 0.05^b^	1.70 ± 0.05^b^	61.27 ± 0.05^ab^	381.57 ± 0.02^a^
8% PPP	17.04 ± 0.05^a^	5.86 ± 0.05^b^	8.67 ± 0.03^c^	12.7 ± 0.05^a^	3.32 ± 0.03^a^	52.41 ± 0.05^c^	311.11 ± 0.03^c^
0.1% PS	15.26 ± 0.03^b^	7.51 ± 0.03^a^	10.54 ± 0.03^b^	2.09 ± 0.03^b^	1.73 ± 0.03^b^	62.87 ± 0.03^b^	376.38 ± 0.03^b^
0.5% CP	15.06 ± 0.05^b^	7.42 ± 0.03^a^	10.45 ± 0.05^b^	2.17 ± 0.05^b^	1.81 ± 0.03^b^	63.09 ± 0.05^a^	376.09 ± 0.05^b^

Values are expressed as mean ± SD of three independent determinations. Values in columns followed by the same letter are not significantly different at p ≤ 0.05 as measured by Duncan’s test.

PPP, pomegranate peel powder; PS, potassium sorbate; CP, calcium propionate.

The high fiber content of PPP is responsible for high moisture (17.04%) and fiber (12.7%) content of muffins with 8% PPP as compared to control and muffins with chemical preservatives. Moreover, the 27% and 20% decrease in fat and protein content was observed in muffins with 8% PPP as compared to control respectively. The decrease in fat per cent is a positive attributes for the muffins as commercial muffins are dense in fat and may responsible for obesity or other life style disorders.

The present findings are in agreement with the results reported by Giri et al.^23^ and Topkaya and Isik^21^, showed an increase in the fiber and ash content with increase in the level of PPP in the muffin formulation.

### Bioactive compounds and In-vitro antioxidant activity of muffins

The effect of addition of PPP and chemical preservatives on bioactive compounds and *In-vitro* antioxidant activity of muffins were evaluated (Table 8) and found significantly higher in muffins with 8% PPP. The total phenols was increased from 0.92 to 12.5 mg GAE/100 g, total tannin from 0.2 to 8.27 mg GAE/100 g, In-vitro antioxidant activity by DPPH from 6.97 to 29.34% and In-vitro antioxidant activity by FRAP from 0.497 to 2.934 mg AAE/100 g in muffins added with 8% PPP. These were approximate 12 times increase in the total phenols and 6 times increase in the In-Vitro antioxidant activity (by DPPH) due to addition of 8% PPP as compared to control. This is obvious due to presence of high level of phenols in PPP (16,364 mg GAE/100 g) as compared to wheat flour (116 mg GAE/100 g)^21^.

**Table 8:** Bioactive compounds and In-vitro antioxidant activity of muffins.

Treatments	Total phenols (mg GAE/100 g)	Total tannin content (mg GAE/100 g)	In-vitro antioxidant activity (%) (DPPH)	In-vitro antioxidant activity (FRAP) (mg AAE/100 g)
Control	0.92 ± 0.05^b^	0.2 ± 0.05^b^	6.97 ± 0.05^b^	0.497 ± 0.05^b^
8% PPP	12.5 ± 0.05^a^	8.27 ± 0.03^a^	29.34 ± 0.03^a^	2.934 ± 0.03^a^
0.1% PS	0.87 ± 0.05^b^	0.3 ± 0.03^b^	5.54 ± 0.05^b^	0.544 ± 0.03^b^
0.5% CP	0.83 ± 0.04^b^	0.4 ± 0.05^b^	5.47 ± 0.05^b^	0.399 ± 0.05^b^

Values are expressed as mean ± SD of three independent determinations. Values in columns followed by the same letter are not significantly different at p ≤ 0.05 as measured by Duncan’s test.

PPP, pomegranate peel powder; PS, potassium sorbate; CP, calcium propionate.

The antioxidant activity of PPP is contributed due to the presence of considerable amount of ellagic acid, punicalagin, ellagic tannins, punicalin etc. as reported by Ismail et al.^11^.

Moreover, there was no statistical difference were observed in the bioactive compounds and In-vitro antioxidant activity of muffins with 0.5% CP and 0.1% PS in comparison with control sample as these are the synthetic preservative which does not have any bioactive.

The increase in the bioactive compounds and antioxidant activity on addition of PPP in muffins were described by Giri et al.^23^; Topkaya and Isik^21^. Similarly, Ranjitha et al.^16^; Ismail et al.^17^ in cookies with PPP and Sulieman et al.^18^ in bread fortified with PPP.

### Scanning images of horizontal section of muffin

**Figure 5:**
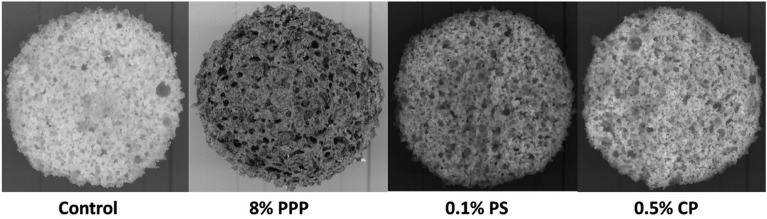
Scanned images of horizontal section of the muffins prepared with pomegranate peel powder and chemical preservative.

The scanned images of the horizontal section of control, muffins with 8% PPP, 0.1% PS and 0.5% CP is depicted in Fig. 5. The actual area of 0.0017 m^2^ and area of scanned image (0.0165 m^2^) for horizontal section of muffins with 8% PPP and chemical preservatives (0.1% PS and 0.5% CP) were considered for the measurement of the total number of pores. The number of pores were based on the visible pores appears on the scanned image of the muffins. The addition of PPP resulted to the decrease in the number of pores (414 no.) in muffins but the number of pores having size larger than 4 mm (139 no.) was found higher than appeared in control and muffins with chemical preservatives. While the number of pores of muffins with chemical preservatives (734 and 809 no.) were almost equal to control sample (703 no.). But, the differences were recorded in the number of pores with size 4 mm and above in each sample of muffins, highest number of pores were observed in muffin with 8% PPP (139 no.), followed by control sample (107 no.), muffin with 0.5% CP (81 no.) and muffin with 0.1% PS (70 no.).

The formation of the large number of tiny gas bubbles in the batter is considered as positive factor for the development of homogenous, uniform pores which leads to the sponginess of the muffins after baking. The entrapment of the air during beating of batter is responsible for the formation of the pores in the crumb of muffin^32^. The addition of 8% PPP in muffin preparation, caused the formation of low number of the large size air bubbles in batter which resulted to conversion into pores with diameter of 4 mm and above as compared to the sample with chemical preservatives.

### Sensory characteristics of muffins

**Figure 6:**
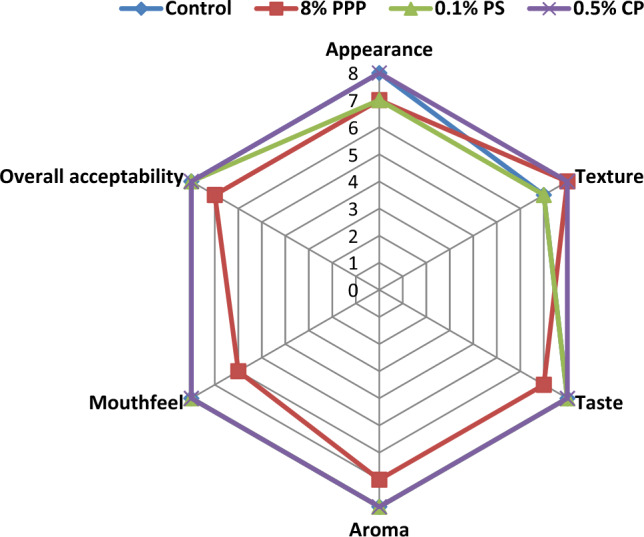
Sensory characteristics of the muffins with PPP and chemical preservatives.

The sensory properties of the muffins with PPP and chemical preservatives were evaluated in terms of the appearance, texture, taste, aroma, mouth feel and overall acceptability (Fig. 6). The positive attributes for the muffin with 8% PPP was the higher score for texture, which is one of the most important product characteristics in case of muffins by the panellist as compared to rest of the treatments. The addition of extra liquid phase in the formulation and moisture holding capacity of PPP resulted to soft texture of the muffins. Moreover, the slightly lower score was received for the taste and mouth feel for the muffins with PPP as compared to control and with chemical preservatives. But, it was more than the liking score, hence overall acceptable. The taste of the muffins with PPP was found slightly bitter as compared to rest of the treatment which might be due to the tannins present in peel. Whereas, the grainy mouth feel was noticed during tasting muffin with PPP. It could be due to the fibrous nature of the peel. Accordingly, the use of dried nuts or dried fruits/ seeds may be suggested to mask the slight bitter taste and to improve the mouth feel of muffins.

The colour of muffins was noticed as cocoa brown due to addition of PPP, and received marginally low score than control which may be due to the presence of natural pigments, granotine present in the peel^52^. It is obvious that, the fortification of any fruit peel by-product in product development will slightly alter the organoleptic properties of the product. Similarly, in present study the addition of PPP slightly alter the properties of the muffins, but it was overall acceptable.

The sensorial attributes of muffins with chemical preservatives such as CP and PS were not significantly affected when compared with control, as these chemicals works as a preservatives rather than to impart any taste or mouth feel or aroma.

Other studies reported that, the use of 15% PPP in muffin cake received lower sensory score than the control but within the range of liking^21^. Muffins with 8% PPP scored overall acceptability within the liking range^23^. Ranjitha et al.^16^ stated cookies fortified with 5% PPP with defatted soy flour was acceptable and Ismail et al.^17^ mentioned about the incorporation of 7.5% PPP in cookies were organoleptically good.

### Storage studies

**Figure 7:**
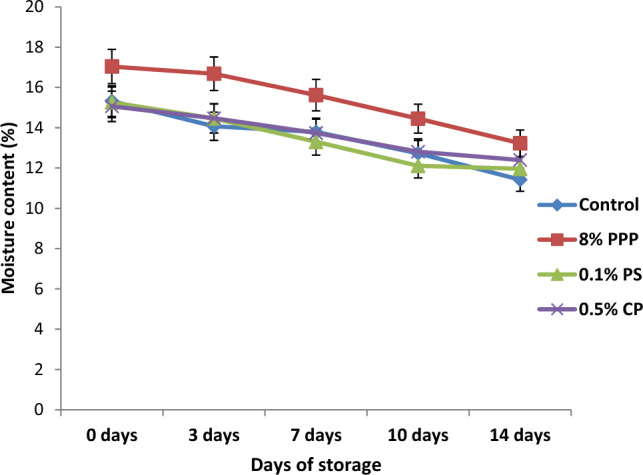
The moisture content of muffins during storage at room temperature.

The moisture content (%), and texture (hardness) of muffins samples were evaluated during storage at room temperature. The moisture content of muffins with PPP and chemical preservatives were decreased during the storage due to the loss of moisture into environment (Fig. 7). The moisture content of muffin with PPP was reduced from 17.04 to 13.23% which was higher than control (15.32–11.42%), muffins with 0.1% PS (15.26–11.96%) and 0.5% CP (15.06–12.4%). It may be due to the higher initial moisture content of PPP added muffins. From the data, it was cleared that, muffins with PPP remained moist for longer period when compared with control sample and muffins with chemical preservatives. It helps to keep crumb of muffins soft and tender which is desirable quality parameter.

**Figure 8:**
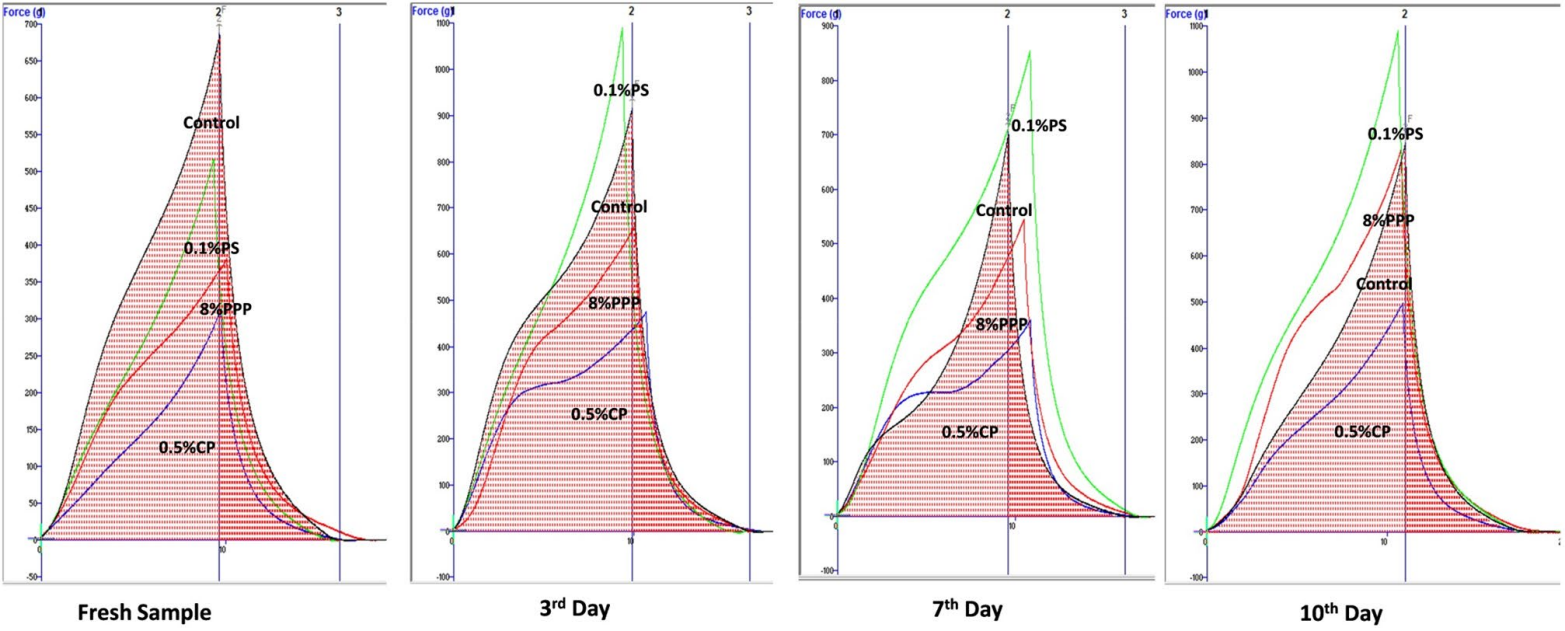
Hardness of muffins during storage at room temperature.

The textural properties (hardness) of the muffin samples were evaluated during storage at room temperature on 0, 3rd, 7th and 10th days (Table 9 and Fig. 8). When the hardness of muffins with 8% PPP was compared with control and sample with chemical preservatives, it was observed that the hardness of sample with PPP (382.12–832.07 g) was higher than the sample with 0.5% CP (313.96–499.86 g), but lowers than control (685.3–844.46 g) and sample with 0.1% PS (517.53–1092.10 g) throughout the storage period. Moreover, during storage, the hardness of individual sample significantly varied. The hardness of all sample on 10^th^ day of storage were significantly higher as compared to hardness recorded in fresh samples. It can be due to the moisture loss from the muffins which resulted to the crumbling texture. The increase in the muffin crumb firmness during storage at room temperature is reported by Soumya et al.^1^. The reason for increase in the crumb firmness may be due to the retrogradation of starch. Similarly, the loss in moisture content leads to increase in the hardness, as water acts as a plasticizer in muffins. The loss of water, responsible for the formation of the cross links between starch and protein^54^. The trend of significant increase in the hardness of all muffin samples on 3rd and 10th day of storage and decline on 7th day of storage were recorded in present study. The similar trend of hardness of cake sample during the storage period of 14 days is reported by Saeidi et al.^55^.

**Table 9:** Hardness (g) of muffins during storage at room temperature.

Treatments	Fresh sample	3rd day	7th day	10th day
Control	685.3 ± 0.5^a^	914.24 ± 0.8^b^	703.90 ± 0.9^b^	844.467 ± 1.0^b^
0.5% CP	313.96 ± 0.7^c^	477.37 ± 1.0^d^	361.01 ± 1.2^d^	499.864 ± 0.7^c^
0.1% PS	517.53 ± 1.0^b^	1092.56 ± 1.1^a^	854.91 ± 0.8^a^	1092.104 ± 0.7^a^
0.8% PPP	382.12 ± 0.2^c^	666.37 ± 0.5^c^	545.54 ± 0.6^c^	832.074 ± 0.5^b^

Values are expressed as mean ± SD of three independent determinations. Values in columns followed by the same letter are not significantly different at p ≤ 0.05 as measured by Duncan’s test.

PPP, pomegranate peel powder; PS, potassium sorbate; CP, calcium propionate.

## Conclusions

The present investigation concluded that, pomegranate peel powder (PPP) is as effective as chemical preservatives such as potassium sorbate and calcium propionate and showed inhibitory action against *Aspergillus* sp. and *Penicillium* sp. in muffins. It may be used as bio-preservative to extend the shelf life of muffins. The use of 8% PPP in muffins significantly affected on the physical properties of muffins batter and baked muffins. The increase in viscosity and specific gravity, with reduction in number of air cells in muffin batter was recorded upon addition of PPP. A firm texture and lower baking loss of baked muffins with PPP was observed as compared to control and muffins with chemical preservatives. The 6 times increase in the fiber content, 14.46% and 18.46% decrease in carbohydrates and energy value was observed in muffins with PPP as compared to control respectively. The developed muffins with PPP indicate high level of bioactive compounds and high antioxidant activities. The sensorial scores for muffins with PPP were within acceptable range. During storage of muffins at room temperature, muffins with PPP revealed high moisture retention, and firm texture.

The PPP has the potential to use as natural preservative with high antioxidant activity and antimicrobial property in shelf life prolongation and functionalization of bakery products where oxidative and microbial stability is important. The limitation of use of PPP is the alteration in the product sensorial properties if used in high quantities. For this, pomegranate peel extract may be studied to use as a natural preservatives in different food product.

## Data Availability

The dataset used and analysed during the current study is available from the corresponding author on reasonable request.

## Abbreviations

PPP: Pomegranate peel powder
PS: Potassium sorbate
CP: Calcium propionate
FSSAI: Food Safety and Standards Authority of India
